# Optimising ePrescribing in hospitals through the interoperability of systems and processes: a qualitative study in the UK, US, Norway and the Netherlands

**DOI:** 10.1186/s12911-023-02316-y

**Published:** 2023-10-11

**Authors:** Catherine Heeney, Matt Bouamrane, Stephen Malden, Kathrin Cresswell, Robin Williams, Aziz Sheikh

**Affiliations:** 1 Centre for Medical Informatics, The Usher Institute, The University of Edinburgh Old Medical School Teviot Place Edinburgh, Scotland, EH8 9AG UK; 2 Usher Institute | Advanced Care Research Centre (ACRC), Usher Institute | Advanced Care Research Centre (ACRC), University of Edinburgh, Edinburgh BioQuarter 9 Little France Road, Biocubes, Edinburgh, Scotland, EH16 4UX UK; 3 Institute for the Study of Science, Technology and Innovation, The University of Edinburgh, Edinburgh, Scotland, UK

**Keywords:** ePrescribing, Interoperability, Integrated system, Data resources, Infrastructure, Electronic health systems

## Abstract

**Background:**

Investment in the implementation of hospital ePrescribing systems has been a priority in many economically-developed countries in order to modernise the delivery of healthcare. However, maximum gains in the safety, quality and efficiency of care are unlikely to be fully realised unless ePrescribing systems are further optimised in a local context. Typical barriers to optimal use are often encountered in relation to a lack of systemic capacity and preparedness to meet various levels of interoperability requirements, including at the *data, systems and services levels.* This lack of *systemic interoperability* may in turn limit the opportunities and benefits potentially arising from implementing novel digital heath systems.

**Methods:**

We undertook *n* = 54 qualitative interviews with key stakeholders at nine digitally advanced hospital sites across the UK, US, Norway and the Netherlands. We included hospitals featuring ‘standalone, best of breed’ systems, which were interfaced locally, and multi-component and integrated electronic health record enterprise systems. We analysed the data inductively, looking at strategies and constraints for ePrescribing interoperability within and beyond hospital systems.

**Results:**

Our thematic analysis identified 4 main drivers for increasing ePrescribing systems interoperability: (1) improving patient safety (2) improving integration & continuity of care (3) optimising care pathways and providing tailored decision support to meet local and contextualised care priorities and (4) to enable full patient care services interoperability in a variety of settings and contexts. These 4 interoperability dimensions were not always pursued equally at each implementation site, and these were often dependent on the specific national, policy, organisational or technical contexts of the ePrescribing implementations. Safety and efficiency objectives drove optimisation targeted at infrastructure and governance at all levels. Constraints to interoperability came from factors such as legacy systems, but barriers to interoperability of processes came from system capability, hospital policy and staff culture.

**Conclusions:**

Achieving interoperability is key in making ePrescribing systems both safe and useable. Data resources exist at macro, meso and micro levels, as do the governance interventions necessary to achieve system interoperability. Strategic objectives, most notably improved safety, often motivated hospitals to push for evolution across the entire data architecture of which they formed a part. However, hospitals negotiated this terrain with varying degrees of centralised coordination. Hospitals were heavily reliant on staff buy-in to ensure that systems interoperability was built upon to achieve effective data sharing and use. Positive outcomes were founded on a culture of agreement about the usefulness of access by stakeholders, including prescribers, policymakers, vendors and lab technicians, which was reflected in an alignment of governance goals with system design.

**Supplementary Information:**

The online version contains supplementary material available at 10.1186/s12911-023-02316-y.

## Introduction

### ePrescribing Implementation

Over the last 20 years, many economically-developed countries have invested substantial resources in the implementation of ePrescribing systems, Computerised Physician Order Entry (CPOE) and Computerised Decision Support Systems (CDSS) in order to improve prescribing processes and patient safety in hospitals [1–4]. Some of the anticipated benefits of combining patient medication data and automated decision support include preventing and minimising the risk of adverse drug events (ADEs) in hospitalised patients and, more generally, improving information access for decision making and patient care [4–8]. Usually, these digital implementations will also require the integration of ePrescribing systems with existing electronic health records (EHRs) as well as with other systems and various data sources (e.g. scheduling, laboratory results, medical imaging, etc.). However, fully realising the benefits of ePrescribing will usually require a process of local optimisation of these systems, so that available functionalities are *fully enabled, appropriately used, integrated* with other relevant health information technologies (IT), *and embedded* with clinical priorities and workflows [9, 10].

This study was conducted as part of a broader programme of research on ePrescribing optimisation [9] and we thus consider *interoperability* as one of the key dimensions of systemic *optimisation*. The thematic framework we used for the broader research programme was that of the medicine use process optimisation lifecycle [9]. Here, we will present the experiences of data sharing goals and interoperability challenges for hospital sites operating in four different national settings: i.e. the UK, US, Norway and the Netherlands. Our aim was to capture how hospitals optimise access to information relevant to the prescribing process, the strategies which have been employed and the impact of these on ePrescribing systems implementation and usage. We consider how hospitals engage with local, regional and national governance and infrastructure to improve interoperability and share data effectively. We argue that achieving data sharing policy goals relies on optimisation strategies that target technical architecture and governance.

## Background

### Integrated care & systemic interoperability

Systems and services interoperability are considered to be critical enablers of integrated care, ensuring that information is effectively transferred across the patient pathway to enable effective oversight of care provision [11, 12]. For example, the integration of IT systems in hospitals with laboratory services and external organisations have identified as key facilitators for the successful embedding of ePrescribing systems [7]. Conversely, the lack of interoperability and integration have also been clearly identified as potential barriers to the acceptability and safe use of ePrescribing systems [5]. Furthermore, systemic errors can also be introduced when transitioning to new ePrescribing systems if there has been a lack of prior attention paid to the integration of data sources and inadequate attention to human factors and underlying work processes [13].

In the early 2000s, the UK NHS Connecting for Health programme envisaged that the *‘ePrescribing systems of the future’* would *‘be developed to offer a more integrated medicines supply chain and more real-time monitoring’* [2]. Cornford et al. suggested that integration should be thought through − and planned for − in relation to the choice of software system and interfacing with existing electronic data resources [2]. While the uptake of ePrescribing systems in secondary care has greatly increased over the last decade, for many hospitals, the focus is now on further optimising these systems to improve safety, efficiency and support patient-facing services [9, 14].

The 2016 report from the National Advisory Group on Health Information Technology in England (the Wachter Report) recommended that planning for further digitisation across the NHS would need to prioritise interoperability via enforcement of standards, targeted funding and penalties [1]. Interoperability is thus considered as the ‘foundation stone’ for improved care, innovation and research. In the US, *the Meaningful Use* EHR program has been renamed *‘Promoting interoperability’* to reflect a greater push for effective communication between systems within hospitals and across different EHR vendors [7]. The European Commission has also made ‘*strong infrastructure and interoperability’* one of three pillars for the creation of a common European Health Data Space [15].

Bates and Samal suggested that interoperability should go beyond simple information sharing to allow for a digital ecosystem where data can be digitally incorporated, manipulated and used by the receiving system [16]. This means aiming for interoperability of systems in order to meet policy aspirations for the possible seamless interoperability of services. Within hospitals, interoperability could be achieved via the purchase of one integrated system or a number of systems, which can then transfer data to each other via data messaging standards and ultimately provide information to an end-user in a readable and usable format via a computer interface [12, 17]. Differences have been found in adaptability to interoperability requirements between large integrated Commercial Off The Shelf (COTS) systems, also referred to as ‘enterprise’ systems [11, 18], and ‘best of breed’ systems, which can require extensive interfacing layers [5]. Both come with advantages and limitations. Integrated systems may avoid the pitfalls of achieving integration and interoperability at the local level and can provide better internal user experience but may also limit customisation for specific workflows [5, 13]. The alignment between work processes and national and technical standards will depend on addressing gaps in infrastructure and governance that impede interoperability [5, 8, 19]. Incentivising hospitals and vendors financially as part of a national program can also provide synergies to interoperability initiatives [1, 20]. Data standards, a single patient identifier and transparent consent or governance structures, can also potentially improve the availability of data for care, cost and resources monitoring, and organisational analytics [11, 12, 20].

We consider the interaction between governance structures and system architecture in relation to optimisation of systems integration or interoperability. Following the Nuffield report *‘Achieving a digital NHS: Lessons for national policy from the acute sector’*, we will refer to interventions targeted at the macro-, meso- and micro-levels, which map onto national, organisational and workflow and technology levels [21]. Governance and infrastructure evolve in response to tensions within the system [22]. Hospitals goals for optimisation of data sharing and systems interoperability arise from tensions or problems arising at all levels of the heterogeneous infrastructural and governance system of which they form a part.

## Methods

### Research ethics committee approval and consent

Ethical approval for this study was granted by the Usher Research Ethics Group (University of Edinburgh) on January 1, 2020 (ref. 1906). Relevant NHS research and development approvals were acquired for UK-based sites on 23/01/2020 (ref.19/HRA/7015). All individual participants were issued with an information sheet and informed consent form prior to the interview. All methods were carried out in accordance with relevant guidelines and regulations The interviews were carried out on platforms approved by the University of Edinburgh and were recorded. All interview transcripts were created by a university approved company; they were then stored without identifying information on a secure platform.

### Sampling

A purposive sampling strategy was used to select cases [23], with information most relevant for our study (see Table 1). This aimed to ensure that we captured those sites, which met the criteria of interest, which included:

- significant experience of digitisation, including different infrastructure at national and local levels;
- sites in different national settings, with different vendors and experience of home grown and ‘best of breed’ approaches; and.
- sites within the Organisation for Economic Cooperation and Development (OECD).

The *’Optimising ePrescribing in Hospitals’* project comprised three phases.

Phase 1 involved a large scale scoping review [9]. Phase 2 – which was conducted in parallel with phase 3 – involved three different focus groups / roundtable events with policy makers and ePrescribing systems users. Phase 3 was a qualitative study and is reported here. Phases 1 and 2 fed into the site selection for Phase 3 to enable us to identify appropriate sites. In each site, we wanted to include a representative sample of professionals involved in ePrescribing, including: clinicians, nurses, pharmacists, IT staff, and service managers (see details in Table 2).

**Table 1:** Site selection criteria

Criteria	Examples	Rationale
**Significant post implementation experience of ePrescribing system**	Healthcare Information and Management Systems Society (HIMSS) / Digitally mature (years since EHR implementation)	Digitally mature sites will have systems which have had time to embed into routine use, and will have gone through multiple iterations of optimisation, offering more opportunities for learning
**Available points of comparison for health system to NHS**	OECD country	Such countries have similar health systems, governance structures, and population characteristics to the UK healthcare context
**EHR system**	Large integrated systems and Best-of-Breed (BoB)	Would allow for the collection of a range of data relating to interoperability and integration optimisations across different system contexts
**Vendor**	A mixture of home grown and commercial off the shelf package providers	Would allow for identification of optimisation strategies specific to issues presented by both system types
**Innovative approach**	For example, integrating genomics and other biomedical data, big data feedback into ePrescribing	Innovation is identified in recent national and international digital infrastructure policies as an important area for development.
**Prior interaction with the site/named contact?**	Gatekeeper/network	Would facilitate recruitment of most knowledgeable and experienced staff.

### Data collection

Approved online teleconferencing platforms, including Teams, Zoom, nhn.no and Skype, were used to carry out our interviews rather than site visits due to the COVID-19 pandemic. This also led to additional delays in organising interviews as hospital staff needed to prioritise the increased care demands that COVID-19 put on the care systems. The challenges associated with COVID-19 resulted in the research team revising the initial study aims in order to be more pragmatic and flexible in terms of the numbers of interviews to be carried out in each site. As a consequence, we had more interviews in some sites than others (see Table 2). Initial contact with the sites were made in early 2020, with the first interviews beginning in May 2020 and the final interview being conducted a year later in May 2021. Two experienced qualitative researchers undertook the interviews (CH and SM), using an interview topic guide, designed by CH (see Supplementary materials). Interviewees were asked about their own role, the ePrescribing history of their hospital, about their EHR system, optimisation and policy context. The topic guide used in the semi-structured interviews is provided as an Appendix in Supplementary material (Suppl 1 / Appendix 1).

We carried out *n* = 54 semi-structured interviews across nine different sites: five sites in the US, two in the UK, one in the Netherlands and one in Norway (see Table 2). Interviews were recorded and transcribed verbatim.

**Table 2:** Site and participant characteristics

	Hospital details			Participant details			
Site	Location	Size	Type	Roles included in sample	Total number	Vendor or home-grown	Integrated or best of breed (BoB)
Site 1	UK	~ 760 beds	Teaching hospital	Pharmacy managers, analysts, pharmacists, nurses, information officers	6	Vendor	BoB
Site 2	UK	~800 beds	Teaching hospital	Pharmacy managers, physicians, analysts, pharmacists, nurses, other ancillary care	13	Vendor	Integrated
Site 3	Netherlands	953 beds	Teaching hospital	Clinical pharmacist, nurses, chief clinical information officer	5	Vendor	Integrated
Site 4	Norway	1,870 beds	Teaching hospital	Pharmacy, physician, nurses, central health I.T clinician	5		
Site 5	US	~ 80 beds	Paediatric Cancer hospital	Pharmacy managers, physicians, analysts, information officers	9	Vendor	Integrated
Site 6	US	~ 800 beds	Teaching hospital	Pharmacy managers, physicians, analysts, pharmacists	8	Vendor	Integrated
Site 7	US	~ 670 beds	Teaching hospital	Physicians, nurses	3	Home-grown	BoB
Site 8	US	~ 1500 beds	Teaching hospital	Pharmacy managers, physicians, pharmacists, information officers	5	Vendor	Integrated
Site 9	US	~ 20,000^a^	Healthcare provider	Informatics and pharmacy leads	2	Home-grown	Integrated

^a^This is distributed across 50+ facilities

### Data analysis

Transcripts were analysed via inductive thematic analysis using NVivo 12 qualitative data analysis software. A coding framework was developed and a sample of the data was coded separately by 2 researchers (CH, SM) and then discussed with the broader study investigative team (AZ, MB) to ensure coding coherence and consistency. Once coding consistency was established through the initial sample coding, data coding was conducted by 2 researchers (CH, SM) with input from the study investigative team. Coding consistency and thematic analysis was subsequently discussed and conflicts resolved at regular meetings of the study investigative team which includes substantial expertise in mixed-methods and qualitative research (AZ, CH, MMB, KC, RW). We used NVivo tools to look for relationships between codes, which allowed us to explore how hospitals optimised by integrating numerous data sources within and beyond the hospital itself. CH carried then out the thematic analysis – with additional input from the research team – which identified 4 key interoperability themes described in the Results section below.

## Results

From the interoperability and data sharing optimisation targets and strategies described by the interviewees in the four national contexts, four distinct key themes emerged.

1. **ePrescribing Interoperability & Patient safety**
2. **ePrescribing Interoperability & Integrated care**
3. **ePrescribing & Care Pathways and Decision Support**
4. **ePrescribing & Services Interoperability**

### ePrescribing interoperability & patient safety

#### Complete medication history & national pharmaceutical record: the example of Norway

The ability to effectively share information about patients tended to be considered in relation to the risk posed by prescribers not having up-to-date, relevant information relating to, for example, patients’ medications and allergies. Furthermore, this can be further complicated when a complete history of a patient medication is not available to the hospital prescribing service, for example, if a patient is taking additional over the counter medication, without sharing this information with medical staff. For example, in Norway prescribers can use the Norwegian Prescription Database (NorPD) [24] to view information gathered by pharmacies, allowing a comprehensive picture of patients’ interaction with the pharmacy via a central medications database. In that context, a prescribing clinician can view not only what colleagues in other care settings have prescribed, but also what the patient has actually picked up from the community pharmacy. In that context, the interoperability requirement is driven by micro factors (the needs of the prescribing clinicians) but can only be implemented via macro actors: i.e. national policy, implementation and resources (NorPD) [24].

*One thing that is something we can see, and now you alter the medication and the electronic medication, but in the recent years we are able to see all medication that this patient has taken out in a pharmacy in Norway, everything. And that’s a very nice thing because of drug overuse and the drug shopping, and the patient says something and you see from the register that they have taken and have gotten medication from several different doctors or something like that. That we can see*.

Site D, neurologist, Norway.

#### Access to pharmaceutical record for emergency care

At a national level, interviewees in the US, Netherlands, Norway and the UK said that it was desirable or necessary to access centralised or shared resources beyond the hospital itself. These resources included drug nomenclature, conditions specific prescribing information and in the Netherlands and Norway some information relating to patients’ medicine history was accessible by relevant professionals within the hospital setting. The following example, in Norway, highlighted the importance of emergency room (ER) medical staff being able to quickly gain access to up-to-date medications information.

*So what we have designed now is one common list that everybody is working in or around. So the doctor will prescribe a medication, an e-prescription, and that will be sent to our central database. And then all the medications will be sent as a list, as a package to the central database. And then when I go to the hospital the doctor in the ER will just click on the button and this list will be loaded down.*

Site D, national IT programme, Norway.

#### ePrescribing interoperability for safe prescribing

In the Netherlands, the G-Standaard is a national central drug data repository containing data across most parts of the medicines pathway. This was useful to prescribers in the Dutch setting due to the breadth of data and functionalities [25, 26]. As well as information on particular patients’ and their healthcare insurance coverage, there was also guidance on the safety of various drug combinations.

*A physician uses the information in the G-Standaard to look up available medicines and prescribe the correct dosage to a patient; a pharmacist uses it to check if the patient’s current medication is compatible with his new one (pharmacovigilance), and to see if his health insurance will reimburse. The G-Standaard makes it possible for invoices to be sent straight to the healthcare insurer; pharmacists, wholesalers and manufacturers use the G-Standaard for placing orders and managing stock.*

Site C, clinical pharmacist, Netherlands

In the above two cases, it was necessary to negotiate interoperability requirements for ongoing access to national level resources with vendors of COTS. In the Netherlands, it was explained to North America vendors that in order to operate in the Dutch health service it would be necessary to ensure that the G-Standaard would remain interoperable with the EHR. In the Norwegian case, the requirement to update the central medicines database (referred to as PLL) was a legal requirement to which any vendor working in the Norwegian setting would be subject [24].

*It would disrupt it but it’s very important. So (Vendor 1) also has to integrate with us. They are also legally…what do you call it? They have to, legally have to send a PLL. They can say oh, we have (Vendor 1) so we can do it. So they have to do a lot of…so we work a lot with (Vendor 1) also. But we call it a health platform, [the region] in the middle of Norway. To make them able to integrate with PLL.*

Site D, national IT programme, Norway.

#### Impact of digitisation on staff communication and work processes

However, interoperability and integration of systems does not necessarily achieve safety objectives, as the following interviewee points out. Due to the apparent availability of information it can become less common for staff to follow up with colleagues involved in different aspects of the patient’s care resulting in the possible loss of a potentially important safety check within the system.

*So, I do think that electronics has totally changed our method of communication now: it’s all on screen and we speak to each other far less. And actually, that then doesn’t give you the opportunity for maybe more junior staff to say, why would give this, and so the nurses can learn from the doctors. I think the doctors go and the information is all there, but notoriously they’re bad at actually looking at different, so they’ll be very familiar with their order screen, their prescribing screen and documentation; they don’t look at the MAR [Medicines Administration Record] very much because they don’t actually administer the medicines*.

Site B, pharmacist safety, UK.

This demonstrates the importance of staff themselves in achieving safety improvements as part of service interoperability and data sharing. The technical infrastructure may be in place but staff are not sure how or are unused to moving between different parts of the system, so that available information is still not being effectively shared.

### ePrescribing interoperability & integrated care

#### Lack of ePrescribing interoperability as a barrier to integrated care

The decision as to which type of system to procure is ultimately taken at the level of the hospital in most of the sites studied (the exception was the site based in Norway, where the EHR system was chosen at a regional level). Findings from earlier work has pointed to system users viewing best-of-breed systems as offering greater flexibility within the hospital to interface with whichever data resources were considered most appropriate [5].

In the following example, this appears to still be partially the case with restrictions on making the required changes to COTS in order to access a diabetes database, with national standards.

*But sometimes the offer wouldn’t always be optimal from [Vendor 2], and so then you had to compromise on that perfect database for the diabetes national standards you can’t use in [Vendor 2] and we were never allowed to have bolt-ons as they called them*.

Site B, lead informatics, UK.

In the above case, this barrier to achieving interoperability with other systems is not a necessary outcome of choosing the COTS system itself, but rather the result of the hospital policy designed to lock-in some of the benefits of the integrated system and avoid too much customisation. In the following quotation, the interviewee takes the view that the best-of-breed legacy systems were in fact not designed to communicate effectively with other systems.

*So interoperability between other systems is challenging but has been made much better with [Vendor 1] as compared to our legacy systems because our legacy systems weren’t necessarily built with interoperability in mind and interoperability was an afterthought.*

Site F, clinical content informatics, US

Here any flexibility offered by the best of breed approach is offset by having an already integrated system designed for ease of information exchange. A user in a site, which is still using the best of breed approach, echoes this sentiment.

*I think the only problem we did have from being quite an early adopter is we tended to go for a lot of best of breeds, which sometimes they have difficulty talking to each other. So, you end up with a lot of isolated systems*.

Site A, ePrescribing Nurse, UK.

#### Health information exchange: sharing of medication record for integrated care

The movement of patients between the hospital and different care settings, poses a risk in terms of miscommunication of medicines information between prescribers in those settings. Being able to follow the patient back to their community doctor or general practitioner (GP) promises significant improvement in terms of the transfer of accurate medicines information. One hospital was involved in a coordinated local initiative with this aim. However, the interviewee describes local care related systems as still not fully interoperable in the sense that the data from GPs could not be directly incorporated into the ePrescribing system and vice versa.

*Health Information Exchange where we can see GP medication lists and allergies and problems added at GP…at primary care level. So we’re able to see that information in secondary care, so it kind of feeds in but it doesn’t necessarily…again it’s on its own page, it doesn’t feed into our patient record, we can’t kind of import it, if you like, it’s there to read only. Obviously, we can’t see anything about the consultations, just things, like I say, problems, diagnoses, allergies and meds.*

Site B, lead pharmacist, UK.

#### Continuity of care and patient safety during clinical handovers

At a micro-level, within a hospital setting a focus on integrating different systems can meet the objectives of patient safety and workflow simultaneously. The following interviewee provides an example, of a patient who had been transferred to a ward following surgery. Here the system was set up to allow access to information on the medication administered in another part of the hospital. This was especially convenient as it avoided the need to contact theatre staff, who may have been busy.

*And you go, oh did they give the patient morphine whilst they were in theatre. I forgot to ask the anaesthetist, or I can’t remember what the anaesthetist said. They can look on the bar…on the system, and it’s there straightaway for them. So you know what patients have had, and what patients are due. So it’s a nice, risk reduction that one.*

Site B, IT midwife, UK.

Access to relevant information, with supporting local infrastructure, helped professionals to manage care and subsequent prescribing when follow-up questions to relevant members of staff and the patient themselves would be difficult. Similarly, technologies supporting specific parts of the care pathway could be redesigned to avoid ADEs arising as a result of busy nurses potentially confusing patients.

In the following quotation, a nurse at a large teaching hospital in the Netherlands eight years on from the implementation of an integrated COTS package. The project described was developed as a pilot to integrate data from Computer on Wheels (CoWs) technology with the EHR system. The technology was designed to recognise different patient specific drawers that can be placed into the CoW and be integrated with the ePrescribing system. This avoids error by providing an up-to-date record of the patient’s medication for prescribers. This hospital developed this pilot with the vendor’s support.

*It’s a pilot and it’s approved and we are now, I think, next month we will implement it on the first two wards. Our idea is that nurses need to work mobile so they have their own mobile workstation and that mobile workstation also includes a small medication box with the drawers of the patients that the nurse takes care of in their shift and with the exchange of shifts they exchange the drawers so that the identifier of the patient is in the drawer in a chip, like a bank. And they can exchange the drawer to another CoW when the CoW recognises the drawer and the nurses on the other shift can work with that same drawer with the same patient.*

Site C, informatics nurse, Netherlands.

Hospitals actively engaged with external partners in order to coordinate aspects of infrastructure and governance to facilitate data sharing. In some cases, hospitals were able to leverage their own position as leaders to negotiate certain aspects of governance, which allowed for optimisations to take place.

### ePrescribing & care pathways and decision support

#### Pharmacogenetic decision support

We studied a US hospital that had considerable scope to develop its capacity in the use of pharmacogenetics data within the prescribing process. Nevertheless, implementation of pharmacogenetics into the CDSS required an overarching strategy at national level. As some form of centralised infrastructure to support this was initially absent the hospital played an active part at the macro level in the Clinical Pharmacogenetics Implementation Consortium (CPIC) funded by the US National Institute for Health to develop the necessary governance and infrastructure.

*And it’s just where the informatics work group and the implementation resources with CPIC come into play, you know, the really fundamental thing that I always keep in mind that you need is you need to take that laboratory result, transform it into a phenotype and then from that phenotype make a clinical recommendation. And so that’s like the really fundamental information translation thing that has to happen and that’s where CPIC, you know, provides these translation tables.*

Site E, chief safety officer, US.

This hospital had a long history of biomedical and especially genetic research, which also placed them in an excellent position to coordinate the CPIC collaboration and negotiate with the lab on receiving data in the most useable format.

At the level of the hospital, the organisation strengths and resources might both demand and facilitate the integration of new systems and sharing of new data. In the following example, pharmacogenetic data were introduced into the CDSS to help tackle the problem of ADEs (micro / meso factor). This involved changing the alert rules to incorporate and reflect the specificity of this type of information. The hospital drew on its own institutional capacities (meso factor) in biomedical research, to negotiate with the vendor (meso factor) and build the system to embed these processes and support new ePrescribing workflows.

… *so there are drugs like mercaptopurine or some of the antidepressants that are affected by two genes, so it’s a two gene pharmacogenetic model, so you have to take into account the phenotype of one gene and the phenotype of the second gene in order to fire the alert. So, you have all these permutations of different combinations that we have to build into the alert rules so that the appropriate recommendations were being fired, based on the patient’s phenotypes.*

Site E, clinical pharmacy and genetics, US.

#### Use of smart medicines pumps

Failures to properly record and administer correct doses were addressed in one case by connecting a smart medicines pump to the EHR, so decisions about infusion rate and concentration were locked into the technology by the prescriber before the nurse interacted with it.

*What’s happening is all the information from the electronic medical record for that heparin is now going into the pump, and all the nurse needs to do now is click and say, yes, I accept, I accept, instead of actually going to their care area, picking the drug, picking the concentration, picking the continuous infusion rate, all that information is going into the pump. That will help reduce our medication errors by when the nurses are incorrectly manually programming the pump, and while it isn’t [Vendor 2] optimisation, it really is part with the technology that we have*,

Site H, manager medicines policy, US.

Our data revealed how hospitals tried to optimise information exchange infrastructure by engaging with infrastructure and governance at macro-, meso- and micro-levels. This was driven by the goals of improving safety, better supporting existing work processes and addressing the actual needs and behaviours of patients.

### ePrescribing interoperability & services interoperability

#### Information exchange vs. integrated pharmaceutical record with decision support

Interviewees pointed out that data could be exchanged / shared electronically between systems, but that did not necessarily mean that this would provide them with immediately useable information [16] as the data provided may not be immediately interpretable by the receiving system. For example, this could happen when some information was provided by one system, such as an electronic referral from primary care to hospital, but where there was subsequently no mechanism to integrate that information directly into the hospital EHR or ePrescribing system as was highlighted for example in the eReferral system implementation described in [27].

Hence, the ability to share data electronically was not considered optimal in terms of efficiency or safety, as it was still necessary for humans to copy data manually into another system, potentially introducing further data entry errors in the process.

*But obviously, patients are out of the area and stuff, do we still need to have mechanisms to post out the information? The information is still just sent out as, you know, just text documents, pdf… There isn’t the ability for a GP to actually import the drug list. We send them in to their system, it’s still got to be transcribed in*.

Site B, lead informatics, UK.

Another example of the limitations of systems interoperability is illustrated in the following example. Despite information from other systems being both importable and viewable, this does not automatically update a central EHR. This means that staff will still need to look for the data elsewhere, potentially logging into other systems to do so.

*…but it’s in a sense a standalone system. We don’t get data into our clinical system from (Vendor 3), they can view information in their system, so they get a view of the labs, and labs can import into their system, but it is not truly interoperable. Like if you enter a patient weight in our ICU [Intensive Care Unit] system it doesn’t go anywhere.*

Site G, nurse informatics, US.

#### The role of governance in enabling ePrescribing services interoperability

The Norwegian Prescription Database (NorPD) has been implemented at a national or ‘macro’ level, with an infrastructure including centrally maintained pharmacy records, which can be accessed by hospital-based staff managing a patient’s care [24]. This was facilitated by a change in the law to allow easier access to the central medications database NorPD. Previously, patients needed to provide explicit consent for information sharing while this is now the case by default and instead patients now need to actively opt-out if they do not wish their records to be accessible and sharable.

*And earlier the doctors had to ask the patient is it okay for you if I look into the RF to look at what the other doctors have prescribed for you? And then the patient would have to say yes, and then you could press the button. But now we have changed one of those laws so that it’s automatic that all doctors are allowed to look into the RF. And then if the patients don’t want you to do it then they have to make a reservation.*

Side D, national IT programme, Norway.

#### Change processes and human factors role in progressing interoperability

Several interviewees talked of the need for data to be interoperable by being readily *usable* by different systems to improve patient safety. This would require not only technical solutions but also a change of well established work processes and habits, such as sending PDFs or using free text for example, which can be read by humans but could not be computed.

*And to be able to do so, the first step is that you need to have the data, and the more free text you have the more difficult it gets. And of course, you have something like natural language processing, but a lot of people dream about that, but the fact is that it’s very difficult to understand the real context of the free text. And in our country at least people use Dutch, English, Greek and Latin, and all kinds of abbreviations, so it’s very hard to reliably distil data from free text.*

Site C, Chief Medical Information Officer, Netherlands.

In some cases, full services interoperability may also require a shared understanding of what is needed in practice. In the following case, interoperability is achieved not just by technical design, but also the willingness of the stakeholders working on the CDSS and in the hospital laboratory services to have a shared understanding of what is required for those needing to use the data in another hospital specialty service.

*…the lab piece is so important at getting the laboratory results because what we hear again and again is, you know, the pharmacogenomic testing is done and it gets returned to the record as a PDF that one cannot build the decision support on. So, that is a huge consideration*.

Site E, chief safety officer, US.

High levels of interoperability are often not achieved due to some of the limitations of infrastructure and governance, in terms of system design, policy support at the macro level, as we have shown in previous sections. However, this also means that interoperability of services has 2 key necessary pre-conditions: the first one is that the technical infrastructure exists for the effective sharing of usable information (i.e. systems interoperability). The second one is for the stakeholders to ensure that they follow the correct steps and processes to ensure that prescribing information is then shared in the way that it is intended. Interoperability of services can not be effectively implemented without these 2 preconditions: i.e. systems interoperability supported by the appropriate human processes necessary for the interoperability of services to operate as it is intended.

## Discussion

### Principal findings

Our research design has enabled some important insights into the strategies and targets for interoperability, as seen from within the hospital across 4 national settings, which we reflect on below. The 4 Key dimensions of interoperability emergent in our study analysis have been summarised in Table 3.

**Table 3:** ePrescribing interoperability key findings

Dimension of Interoperability	Rationale	Examples
**1. ePrescribing Interoperability & Patient safety**	Interoperability is considered as an enabler of increased patient safety, for example, to ensure that a list of current prescribed medications is known to a new prescriber to avoid potential issues with polypharmacy or that a list of current medication is available during emergency care.	National Pharmaceutical Record (G-Standaard) in the Netherlands provides a complete Prescribing History for the treating medical team (community or hospital based)
**2. ePrescribing Interoperability & Integrated Care**	Systems and services interoperability is considered to be a critical enabler of integrated care as a means of ensuring that information is effectively transferred and shared across the patient pathway and enabling effective oversight of quality and efficiency of care provision.	The effective integration of HIT systems in hospitals with laboratory services and external organisations have been found to be key facilitators in the successful embedding of ePrescribing systems. Conversely, issues of interoperability and integration have recurrently been identified as potential important barriers to the acceptability and safe use of ePrescribing systems (5).
**3. ePrescribing Interoperability, Care Pathways & Decision Support**	Interoperability can be used as a mean to optimise local integrated care pathways to suit local care priorities / protocols and develop custom decision support systems	One US hospital used its own institutional capacities in biomedical research to develop local pharmacogenetic rules and then negotiated with the vendor to implement the pharmacogenetic rules in the ePrescribing system to support new prescribing process and workflows.
**4. ePrescribing for full services Interoperability**	In this scenario, interoperability goes beyond the simple sharing of data across different clinical systems and instead allows for the information to be usable by the end-users for the specific healthcare purpose at hand in a variety of settings & contexts. Systems and stakeholders need to work in synch to ensure that full services interoperability is achieved.	National Pharmaceutical Record (NorPD) can be used in a variety of settings (e.g. pharmacy, primary or secondary care) and for a variety of purposes (e.g. routine care, emergency care, research and public health) This is made possible by national legislation which enables patient data to be shared in a variety of context for a variety of purposes.

### Safety as a key interoperability driver

Safety remains the key driver for optimisation strategies aimed at improved data sharing within hospitals and with external care settings and resources. Safety drivers for increased systems interoperability vary widely and include the goal of monitoring patients’ behaviour in terms of picking up prescriptions, including new information to mitigate risks of poor response to medication and integrating smart technologies to the EHR to minimise staff input and therefore scope for errors. These involve staff and hospitals developing strategies to gain access to resources at the macro-, meso- and micro-levels.

The sites for our study shared the goal of integrating and sharing different types of data across various technologies, systems and databases in order to achieve care related goals. However, how well they were able to achieve interoperability with external sources or integration with internal data systems depended on context.

Access to national level resources can require a national strategy when negotiating with vendors. In the Norwegian case, this was facilitated by a change in the law facilitating prescribers’ access to a central medications database (macro factor). This infrastructure is managed centrally at a national level but is impactful at hospital level [24, 28]. In the case of Norway, changes to the law (macro factor) backed up the commitment to make sharing easier by replacing an opt-in model for patients allowing access to the central medicines database, with an opt-out. Most hospitals we spoke to had the freedom and responsibility to negotiate with the vendor directly to ensure the hospital ePrescribing system was interoperable with national level resources.

In one case in the US, the hospital itself was very proactive in developing guidelines for pharmacogenomics. This same hospital was able to draw on a long history of biomedical research, where relationships between lab and clinic had been fostered. Such relationships may be difficult to generalise, nevertheless they are a useful benchmark for what innovations can be achieved in particular areas, such as pharmacogenetics, given a particular configuration of skills, history and motivations.

Above, we have examined strategies and impacts of interoperability optimisations into macro, meso and micro levels. Our data supports the insight that being part of a heterogeneous data assemblage can be both generative and constraining [22]. Our unit of analysis was the hospital at a meso-level, however, it is clear that the macro and micro are impactful on interoperability optimisations. Lack of adequate standardisation across different hospitals in a national or international setting are barriers to incorporating data into the CDSS at local level. Similarly, reluctance to enter or share data by professionals used to specific and non-digitised ways of working can limit interoperability.

The Netherlands site indicated that they were able to leverage national governance to persuade the vendor to maintain interoperability with a key national data resource. Integration of data resources curated nationally was promoted via provision of resources, which were seen in the Netherlands and Norway as of great value to prescribers. In the Netherlands and Norway, hospital staff and those working at a centralised level, explained how they had been very clear with vendors that this access must be facilitated if the vendor was to function in that national setting. The creation of a national approach to interoperability is perhaps easier in smaller population size countries. Norway, for example has about five million inhabitants compared to the UK’s 67 million, across 4 nations, each with its own distinct national health system and eHealth infrastructure. However, it is also the case that both Norway and the Netherlands have historically had a structured and uniform approach to public health records governance with a less heterogeneous population and health system than countries like the UK and US [29].

Removing barriers to interoperability requires a robust approach to governance and infrastructure. We noted different priorities for interoperability. In some cases, a decision to avoid over-customisation of an integrated system can be taken at the hospital or trust level in the UK, but this can also mean that the hospitals may then be unable to access useful national resources.

There was evidence that integrated systems are perhaps perceived as a safer base for achieving seamless interoperability, whereas in the past, the flexibility offered by best of breed systems may have been an important factor for implementation [5]. For some hospitals, there was a sense that they had or were still hampered by adherence to a best of breed model wherein interoperability was only considered as an afterthought. Most of the hospitals in our study worked with COTS but had different interoperability strategies and targets depending up on factors such a national tradition of sharing health and prescribing data or relationships between different parts of the hospital and the hospital’s own policies. Policy changes in the US to the Meaningful Use Act are planned in terms of encouraging vendors of all types to look beyond the needs of their particular client base to ensure that systems supplied by different vendors can also communicate. In other national contexts, there are existing clear parameters on interoperability and data access, which vendors are made aware of when entering that market.

### Strengths and limitations

This thematic analysis of ePrescrining interoperability was conducted by a team of experienced qualitative researchers (CH, SM, KC, RW) and mixed-methods researchers (AZ, MMB) and consistency to the thematic framework was ensured through regular quality assurance discussions throughout the project. Our research design has enabled us to examine a range of hospitals – some using the same ePrescribing solutions or enterprise systems – operating in different national policy contexts; furthermore, we also included two hospitals using a best-of-breed approach. Selecting hospitals with a significant history of digitisation has enabled us to capture, in some cases, hospitals with experience of using both best of breed and integrated systems approaches.

The limitations of our study include that our cross-sectional multi-site approach means that we were not able to follow-up at different time periods to gain further insight into interventions evolutions over time. We were not in a position either to study in-depth the interactions and negotiations between the macro-, meso- and micro-level changes to infrastructure and governance. The number of sites with integrated systems also far outnumbered those working with a best of breed approach. This was to some extent an artefact of our sampling criterion, whereby most of the sites recommended as being advanced in their digital journey had an integrated system by the time of our fieldwork. However, as previously noted, a number of the sites that had integrated systems had moved from a previous best-of-breed approach and were hence able to reflect and share these implementation experiences with our research team. Finally, there is greater variation in the numbers of interviews gathered in particular sites due both to the challenges of identifying suitable stakeholders at these sites during a severe public health crisis which added delays and restrictions due to the COVID-19 pandemic. This resulted in the overrepresentation in the data of UK and the US interviewees compared to the other two European cases (Norway and the Netherland).

### Interpretation in the light of the wider published literature

Previous work has referenced a lack of national or international standards as potentially hampering efforts to incorporate data resources into the CDSS [5]. Several sites discussed the need for standard terminologies such as offered by SNOMED CT [20, 29, 30]. In countries such as the Netherlands and Norway, a national level culture of sharing public records over time has established data standards, which can potentially be built upon. Indeed, one of the acknowledged achievements of the National Programme for IT was to establish a standardised and unique NHS patient identifier [1] which is critical for aggregating patient records held in different systems for an individual patient [31]. In turn, this can be used to create a single virtual patient electronic health record using clinical portal technology as described for example in [32].

The decision of hospitals with regards which systems to choose – integrated or best of breed, home grown or COTS – had implications for how successful they would subsequently be in accessing data resources. It was perhaps perceived historically that best-of-breed systems could provide greater flexibility in terms of customisation to local preferences and protocols compared to enterprise systems. However, there seems to have been a shift more recently in giving more credits to enterprise systems as providing a more streamlined and manageable means to achieving organisational interoperability aims with less flexibility for individual services becoming a necessary trade-off to that end [5].

### Implications for policy, practice and future research

There is now substantial knowledge on the range of approaches to optimise and potentially enhance the use of hospital ePrescribing systems [5, 9]. Optimisation strategies can be powerful, achieving strong clinical buy-in and ownership, while also allowing ePrescribing systems and workflows to be customised extensively to local clinical and specialty-specific needs [33]. Although localised innovation may be an effective method to improve usability and relevance of ePrescribing systems, optimisation and interoperability at scale will be dependent on success stories being cascaded and efficiently applied elsewhere. Poorly managed localised customisation has the risk of leading to increasingly divergent systems and workflows, making policy deliberations and large-scale interventions difficult to manage [9, 34].

Policy-focused interventions will need to strike a balance between being sensitive to local needs, while delivering interventions that can drive tangible improvements in clinical outcome measures across large patient populations [34]. Further comparative work on infrastructure and governance in different national settings could shed additional light on the role of centralised leadership and negotiations with vendors. A longitudinal aspect to such research would enable a greater insight into the impact of various interventions to optimise systems interoperability.

## Conclusions

Hospitals are part of a larger architecture, with multiple levels of governance and infrastructure. Within this wider assemblage upward causality enables new unexpected capacities to emerge. The downward causality constrains and limits the possibilities for further interconnection opportunities [22]. When attempting to optimise ePrescribing via improved interoperability, hospitals were both enabled and constrained by existing internal and external infrastructure. These constraining and enabling factors were visible in how hospitals interacted with vendors when establishing access to data resources. At the local level of the hospital, many sites reported the value – in terms of interoperability – of an already integrated system, which has, at least hospital wide interoperability, as part of its model. There appears to be a shift in thinking, as vendors in specific national settings embrace context-dependent requirements for accessing data held on different systems to be accessible via the ePrescribing system. In some cases, there have been a national push to establish policy and architecture to support ePrescribing by providing useful and usable data resources.

Staff at the hospital level, frequently saw it as their job to negotiate safety goals via interoperability and data sharing with vendors at micro and macro levels, as well as organisational of meso level. Vendor willingness to support making the necessary adjustments to the ePrescribing system appears to be based on a number of factors, which include a national culture of providing and using shared resources and a strong hospital culture of innovating in technology and data. The sophistication of integration and interoperability strategies varied with particular targets playing to both the expertise and national settings of our sites.

It is clear that achieving full services interoperability across all parts of the system or assemblage, including ePrescribing, will remain an aspiration given that ensuring interoperability in one part of the overall system can mean sacrificing it in others. Improved interoperability remains a pervasive goal in terms of optimisation of ePrescribing, especially where it appears to increase safety and support workflow. Hospitals found themselves as part of a complex system in which it was necessary to take into account data sources, provided at national, organisational and by particular technologies.

### Supplementary Information

**Additional file 1: Appendix 1.** Optimisation of ePrescribing in Hospitals topic guide

## Data Availability

The datasets generated and/or analysed during the current study are not publicly available due to the nature of the ethical approval granted for this study.
