# Supplementary material for: Optimising ePrescribing in hospitals through the interoperability of systems and processes: a qualitative study in the UK, US, Norway and the Netherlands
Source: BMC Med Inform Decis Mak. 2023 Oct 11;23:211. doi: 10.1186/s12911-023-02316-y (PMC10568858; doi:10.1186/s12911-023-02316-y)
Supplement: Supplementary file 1 — Additional file 1: Appendix 1. Optimisation of ePrescribing in Hospitals topic guide [file 12911_2023_2316_MOESM1_ESM.docx]

**Appendix 1**

**Optimisation of ePrescribing in Hospitals topic guide**

| **Preamble: We want to hear your experiences and views on what has happened subsequent to the implementation of your ePrescribing (Hospital Electronic Prescribing and Medicines Administration [HEPMA], CPOE) system. We have prepared the questions below to guide the conversation and ensure we cover the relevant topics with you.** |
| --- |
| 1. **Descriptive/Factual Information** |
| **1.1 About the interviewee** |
| - What is your professional background? - What is your role within the organisation? - What do you understand to be optimisation of ePrescribing? |
| **1.2 Organisation** |
| Tell us about the organisation |
| - History of digitisation - How has this shaped the ePrescribing route taken - What have been the barriers/facilitators? - What did you learn from this experience? |
| **1.3 System** |
| Can you tell me a bit about your current EHR system? |
| - What is it?   - Vendor   - BoB/Overarching system - Tell us about the implementation process   - Especially if/ how it impacted on ePrescribing. - What systems have been involved in optimisation of ePrescribing?   - CDSS/(CPOE?)   - Lab systems |
| **2. Optimisation** |
| 2.1 How do you understand optimisation? |
| - Do you think there was a baseline?   - Is it clear where implementation of the system ends, and optimisation begins?   - Is there a before and after optimisation? |
| 2.2 Tell us about what improvements to the ePrescribing system have made in your organisation |
| - Please describe some of the work you have been doing that falls under optimisation.   - Customisation   - Addition of new information sources   - Integration of systems   - Staff deployment |
| 2.3 What impacts have been noted? |
| - - Examples?   - How were these measured? - Which factors have most influence on practice and use in ePrescribing? - Who besides the interviewee is involved in the management, design and use of current ePrescribing/ Electronic Prescribing and Medicines Administration (ePMA)/CPOE? - What roles do they have in optimisation? |
| **3. Policy context** |
| 3.1 What is the policy background/drivers? |
| - ePrescribing specific - General policy changes that have motivated improvements in the ePrescribing process/system - Country specific/international/specialisation |
| 3.2 What other drivers for optimising ePMA/ePrescribing? |
| - Internal/External   - Safety   - Audit   - Cost effectiveness |
| 3.3 Opinion about governance of optimisation process |
| - What have you learned? - What do you wish you were aware of when you began the optimisation process? |
| 3.4 What changes should be made to current guidance/governance systems to smooth the optimisation process? |
| **4. Have we missed anything?** |
| As the experts on your own experiences and views – do tell us if there is anything we have not covered that you think may be important to developing a complete view of optimisation of ePrescribing? |
